# Adsorption of Fluoroquinolone Antibiotics from Water and Wastewater by Colemanite

**DOI:** 10.3390/ijerph20032646

**Published:** 2023-02-01

**Authors:** Gül Gülenay Hacıosmanoğlu, Marina Arenas, Carmen Mejías, Julia Martín, Juan Luis Santos, Irene Aparicio, Esteban Alonso

**Affiliations:** 1 Environmental Engineering Department, Faculty of Engineering, Marmara University, Uyanık Cd. No: 6, Istanbul 34840, Turkey; 2 Departamento de Química Analítica, Escuela Politécnica Superior, Universidad de Sevilla, C/Virgen de África, 7, E-41011 Seville, Spain

**Keywords:** fluoroquinolone antibiotics, colemanite, adsorption, water, wastewater

## Abstract

Pharmaceutical residues in water and wastewater have become a worldwide problem with environmental and public health consequences. Antibiotics are of special importance because of the emergence of antibiotic-resistant genes. This study evaluates the adsorptive removal of four common fluoroquinolone antibiotics by using natural colemanite as an alternative adsorbent for the first time. Batch adsorption experiments were conducted for the mixture of fluoroquinolones as well as for individual compounds during the isotherm studies. Adsorption kinetic results indicated that the process followed the pseudo-second-order (PSO) model, while the Langmuir model described the sorption isotherms. The effects of pH and temperature on adsorption performance were determined, and the results indicated that the adsorption was endothermic and spontaneous, with increasing randomness at the solid–liquid interface. The effects of real water and wastewater matrices were tested by using tap water, surface water, and wastewater samples. Reusability experiments based on five adsorption–desorption cycles indicated that the adsorption performance was mostly retained after five cycles. The adsorption mechanism was elucidated based the material characterization before and after adsorption. The results indicate that colemanite can be used as an effective and reusable adsorbent for fluoroquinolone antibiotics as well as for other pollutants with similar physicochemical properties.

## 1. Introduction

Pharmaceuticals in water bodies have become a major concern worldwide with the advances in analytical chemistry in recent decades. The occurrence of residual antibiotics in environmental matrices is a serious case of pharmaceutical pollution, with hazardous effects on ecosystems and public health. Antibiotics are of particular importance because of the potential overgrowth of resistant organisms. These pharmaceuticals are released into the environment via human and animal metabolic waste, agricultural runoff, and industrial antibiotic waste [1].

Conventional treatment plants are not able to completely remove antibiotics since these systems are designed to treat macro pollutants [2]. As a result, untreated antibiotic residues are released into the environment. As indicated in a recent review, antibiotics concentrations in the aquatic environment vary significantly (up to 21,400 ng/L in wastewater effluent) and depend on different factors, including seasonal variation, prescription, and wastewater treatment plants’ operating procedures [3]. Moreover, the physicochemical properties of the antibiotics, including water solubility, log octanol–water partition coefficient (K_OW_), adsorption coefficient (K_d_), and dissociation constant (pK_a_), affect their chemical behavior and fate in the aquatic environment [3]. These substances, even at trace concentrations, cause threats to the health and safety of aquatic life. Moreover, cumulative and synergistic effects of different types of antibiotics in the water bodies can lead to augmented environmental hazards [4]. In addition to the direct environmental impacts, increased bacterial resistance is an important effect of antibiotics in water bodies [5]. Regarding these concerns, more efficient treatment processes for the removal of antibiotics from wastewaters are being evaluated, such as membrane filtration and advanced oxidation and adsorption processes. Each process has its own advantages and disadvantages. Among different alternatives, the adsorption process is a promising solution with simple operation, relatively low cost, and high efficiency without the formation of toxic intermediate byproducts [6,7].

This study focuses on the adsorptive removal of fluoroquinolones, which are among the most widely prescribed antibiotics. The occurrence of fluoroquinolone antibiotics has been reported in the samples from wastewater influents and effluents, soil and sludge, as well as surface water and groundwater [3,8,9]. In the literature, different sorbent materials have been tested for the removal of fluoroquinolones from aqueous solutions, for example, biochar-based composite materials [10,11], magnetic nanoparticles [12], clay-based materials [13,14], alpha alumina nanoparticles with surface modification [15], nanofiber sorptive membranes [16], and reduced graphene oxide MoS_2_ heterostructures [17]. However, research is ongoing for alternative adsorbents with higher efficiency, stability, good reusability, and lower cost.

In this study, colemanite with the formula of Ca_2_B_6_O_11_·5H_2_O is utilized for the adsorptive removal of four common fluoroquinolones (ofloxacin, norfloxacin, ciprofloxacin, and enrofloxacin). To the best of our knowledge, this study is the first to evaluate the natural colemanite mineral as an adsorbent material. The adsorption studies were applied to both individual and mixed antibiotic solutions to assess the competitive effects between the antibiotics. Isotherm and kinetic studies were conducted, and pH and temperature effects on the adsorption were determined. Studies with real water and wastewater samples were also conducted to reveal the matrix effects on adsorption capacity. Adsorbent reusability was tested by conducting five consecutive adsorption–desorption cycles. Finally, to determine the adsorption mechanism, the material characterization results were compared for colemanite before and after fluoroquinolone adsorption.

## 2. Materials and Methods

### 2.1. Materials

The analytical standards were provided by Sigma-Aldrich (Steinheim, Germany). HPLC-grade solvents and analytical-grade reagents were obtained from Panreac (Barcelona, Spain) and Scharlab (Barcelona, Spain). Initially, accurately weighed amounts of enrofloxacin (CAS No: 93106-60-6), ofloxacin (CAS No: 82419-36-1), norfloxacin (CAS No: 70458-96-7), and ciprofloxacin (CAS No: 85721-33-1) standards were diluted in methanol/formic acid mixture (0.5% *v:v*) to prepare 1000 mg/L stock solutions. The stock solutions were diluted with deionized water to prepare individual standards. Antibiotic mixtures were also prepared by mixing and diluting the individual standard solutions. Colemanite (CAS No: 1318-33-8) was supplied by the Etimaden General Directorate (Ankara, Turkey). Prior to use, it was washed with dilute HNO_3_ (0.1 M), rinsed with deionized water and 0.01 M Ca(NO_3_)_2_, and sieved with a 200 mesh sieve to obtain purified and uniform colemanite.

### 2.2. Experimental Methods

Adsorbent characterization was carried out by X-ray diffraction (XRD), specific surface area (SSA), scanning electron microscopy/energy-dispersive X-ray spectroscopy (SEM/EDS), zeta potential, and Fourier transform infrared spectroscopy (FTIR) analyses for the colemanite samples before and after adsorption process. All characterization analyses were performed at CITIUS laboratories (University of Seville, Spain).

SEM/EDS analyses were conducted using a FEI-TENEO scanning electron microscope (FEI Ltd., Hillsboro, OR, USA), with energy-dispersive X-ray spectroscopy for microanalysis. FTIR analyses were performed with a Tensor II spectrometer (Bruker Optics Inc., Karlsruhe, Germany) by applying the KBr pellet technique. The spectral range was 4000–400 cm^−1^, and the spectral resolution was 4 cm^−1^. XRD analyses were performed with a Bruker D8 Advance A25 diffractometer (Bruker, AXS GmbH, Karlsruhe, Germany) with a Cu Kα radiation source (40 kV, 30 mA). Diffractograms were measured in the 2θ range of 1–70° with a step size of 0.03° and a step time of 0.1 s. SSA, and the pore volume of colemanite were assessed by nitrogen adsorption and desorption at 77 K using an ASAP 2420 gas sorption analyzer (Micromeritics Instruments, Norcross, GA, USA) after degassing at 373 K for 3 h. For the zeta potential analyses, colemanite suspensions (0.1 g/L) were prepared in water by adjusting the pH at different values using 0.1 M HCl or NaOH and equilibrated by mixing for 24 h. The pH of the suspensions was measured with a BASIC 20 pH meter (Crison Instruments, Barcelona, Spain). The zeta potential of the prepared suspensions was determined at 25 °C by a Zetasizer Nanosystem system (Malvern Instruments, Southborough, MA, USA).

Batch adsorption experiments were conducted using a 10 mL aqueous solution of the fluoroquinolone antibiotics. The colemanite dose was 1 g/L, and control samples were prepared without colemanite addition. The following conditions were applied during the adsorption experiments unless otherwise specified. The initial adsorbate concentration was 10 mg/L, the contact time was 24 h, and the temperature was 25 °C. The samples were mixed at 350 rpm using a multi-stirrer magnetic device (J.P. Selecta S.A., Barcelona, Spain). After the adsorption process, the samples were filtered by 0.2-micron filters, diluted in deionized water, and analyzed by LC-MS/MS. The effects of different conditions on the adsorption performance were evaluated by changing one variable at a time while keeping the other variables constant, as specified above. For the kinetic studies, different contact times (5 min to 7 days) were applied. For the isotherm experiments, different initial antibiotic concentrations were used, between 1 to 200 mg/L. In these studies, a wide concentration range was selected in order to visualize complete adsorption isotherms [18].

Thermodynamic parameters were determined by conducting the adsorption experiments at different temperatures (15, 25, and 35 °C). The effect of solution pH was evaluated by conducting the adsorption experiments in solutions with different pH values (between 2 and 11), adjusted by 0.1 M HCl or NaOH. The effects of real water and wastewater matrices were determined by performing the sorption experiments using tap water, surface water, and wastewater samples. Surface water samples were from the Guadalquivir River (Seville, Spain), and samples of tap water were collected within Seville city. Effluent wastewater samples were obtained from a wastewater treatment plant located in Seville (Spain). The samples were stabilized with acetonitrile (0.5% *v/v*) and stored at 4 °C until analysis. Before the experiments, samples were filtered by using a 1.2 μm glass fiber filter (Whatman Inc., Maidstone, UK) for the removal of suspended material.

For desorption and reuse experiments, the samples were centrifuged (200 rpm, 5 min) after the adsorption experiment. The liquid phase was discarded, and 10 mL of 0.1 M NaOH was added to the solid phase. Centrifugation was applied again, followed by rinsing with deionized water, centrifugation, and drying. The obtained colemanite after adsorption was used for the next adsorption experiment. Five adsorption and desorption cycles were applied, and a change in the adsorption capacity was calculated to assess the reusability of the adsorbent. The experimental conditions used in the adsorption and desorption studies are listed in Table S1 (Supplementary Materials).

Qualitative and quantitative analyses of the target compounds were performed by liquid chromatography tandem mass spectrometry (LC-MS/MS) using the analytical conditions previously described [19,20]. For this purpose, an Agilent 1200 series HPLC coupled to a 6410 triple quadrupole mass spectrometer (MS) equipped with an electrospray ionization source (Agilent Technologies, Lexington, MA, USA) was used. A Kinetex Polar C18 (50 × 3.0 mm, 2.6 μm particle size) column (Phenomenex Co., Torrance, CA, USA) was used for chromatographic separation. The mobile phase was a binary gradient mixture of 10 mM ammonium formate in water with 0.05% formic acid (solvent A) and methanol (solvent B). LC-MS/MS parameters for the target antibiotics are described in Table S2.

### 2.3. Data Analysis

The adsorption studies were done as duplicate experiments. Data points in the figures show the mean values, and the error bars represent the standard deviation. The amount adsorbed by the unit weight of colemanite is calculated by Equation (1).

(1) $$q_{t}=\frac{\left(C_{0}-C_{t}\right)V}{m}$$

where q_t_ is the amount of fluoroquinolones adsorbed (mg/g) at time t, C_0_ is the initial adsorbate concentration (mg/L), C_t_ is the adsorbate concentration at time t (mg/L), V is the volume of the solution (L), and m is the weight of the adsorbent (g).

Kinetic and isotherm models applied to the experimental data are given in Table S3. In order to determine the model parameters, nonlinear optimization by Excel Solver was applied, as described in a previous study [18]. Kinetic and isotherm model fit results were assessed by calculating the normalized root mean square error (NRMSE), chi-square (χ^2^), and coefficient of determination (R^2^). The parameters used for model evaluation are shown in Table S4.

## 3. Results and Discussion

### 3.1. Adsorbent Characterization

The SSA of colemanite, obtained by the Brunauer, Emmett and Teller (BET) method, was 1.41 m^2^/g. The obtained nitrogen adsorption–desorption isotherm of colemanite (Figure S1) can be classified as type IV according to the IUPAC definition, indicating a mesoporous structure with an H4 hysteresis that is common for aggregated crystals [21]. The pore volume and average pore size of colemanite were determined by the Barrett, Joyner, and Halenda (BJH) model. The average pore diameter was found to be 25.33 nm, and the pore volume was 6.46 × 10^−3^ cm^3^/g. Since the pores widths between 2 and 50 nm are classified as mesopores based on the IUPAC definition [21], colemanite can be categorized as a mesoporous material.

The surface morphology of colemanite was visualized by SEM, and the chemical composition was assessed by EDS analysis. The SEM image and EDS analysis of colemanite are shown in Figure S2a and b, respectively. In the SEM image of colemanite, both macropores (>50 nm) and mesopores (2–50 nm) can be observed. According to the EDS results, the surface composition mainly includes B, O, and Ca elements in accordance with the colemanite structure, and trace amounts of Si, Mg, and C are also observed, which can be caused by minor impurities.

The results of the XRD analysis of colemanite are given in Figure S3. These results indicate that the amorphous percent of the sample was about 15.5%, and the crystalline phase consists mainly of colemanite (89.0%) as well as traces of calcite (9.5%) and quartz (1.5%). FTIR analyses and zeta potential measurements at different pH values were also used for the adsorbent characterization, which will be discussed in the adsorption mechanism part, together with the characterization results of colemanite after adsorption.

### 3.2. Kinetics and Isotherm Studies

**Figure 1:**
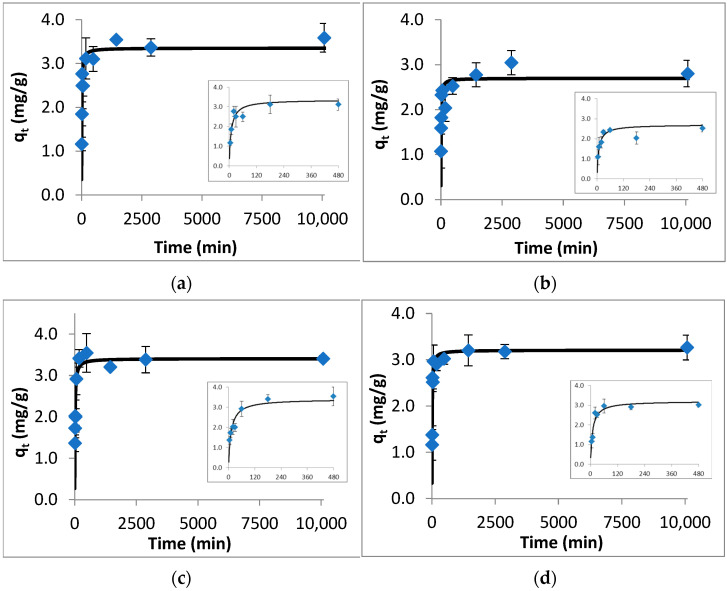
Kinetic data (points) and PSO model fit (solid line) for the adsorption of fluoroquinolones by colemanite. (**a**) Ofloxacin, (**b**) norfloxacin, (**c**) ciprofloxacin, (**d**) enrofloxacin.

The results of batch adsorption kinetic studies are given in Figure 1. From the obtained results, it is observed that for each target compound, the amount adsorbed per unit weight of colemanite increased quickly within the first 480 min and then reached equilibrium gradually in 24 h. In order to allow us to discriminate the mass removed as a function of time, a window zooming in on data between 0–480 min is shown in Figure 1. The kinetic model fit results (including the PFO, PSO, and Elovich models) are given in Table S5. Based on these results, the PSO model describes well the experimental data with the lowest NRMSE and χ^2^, with R^2^ close to unity. (For enrofloxacin, both PFO and PSO models fitted well to the kinetic results.) Though the PSO model is usually linked to chemical adsorption, this assessment should be supported by the other analyses (including material characterization), which are discussed in the adsorption mechanism part.

**Figure 2:**
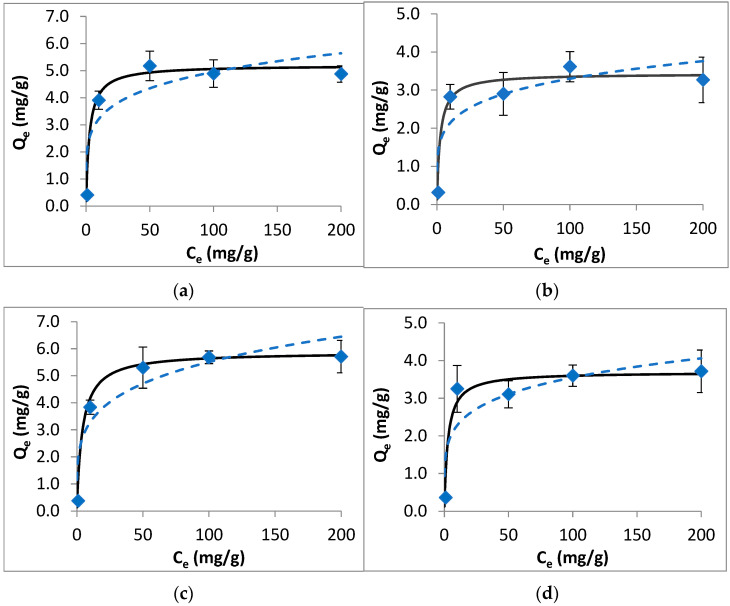
Isotherm data (points), Langmuir model (solid line), and Freundlich model (dashed line) for the adsorption of fluoroquinolones by colemanite. (**a**) Ofloxacin, (**b**) norfloxacin, (**c**) ciprofloxacin, (**d**) enrofloxacin.

The results of equilibrium studies, along with the isotherm model fit, are shown in Figure 2. The obtained data were modeled using Langmuir and Freundlich equations. The isotherm model fit results (including the calculated model parameters and statistical measures for the applied models) are given in Table S6. For each target compound, the Langmuir model fits better to the equilibrium data than the Freundlich model, with high R^2^ and low χ^2^ and NRMSE values. While the Langmuir model describes monolayer adsorption with a homogeneous surface and energy on the adsorbent surface, the Freundlich model expresses multilayer adsorption with heterogeneous energy distribution of the active sites. In this regard, the obtained results indicate the energetically homogeneous surface of the colemanite with the monolayer adsorption of fluoroquinolone antibiotics on it. The highest maximum Langmuir adsorption capacity (Q_max_) of colemanite was 5.88 mg/g for ciprofloxacin, followed by ofloxacin, enrofloxacin, and norfloxacin with the Q_max_ values of 5.19, 3.69, and 3.43 mg/g, respectively.

Another important parameter for the Langmuir model is the separation factor (R_L_), which is used to check if the adsorption process is favorable or unfavorable (Equation (2)).

(2) $$R_{L}=\frac{1}{1+K_{L}C_{0}}\;$$

where C_0_ and K_L_ are the highest initial adsorbate concentration and the Langmuir constant, respectively.

From the results of equilibrium experiments, R_L_ values were determined as 0.013, 0.012, 0.020, and 0.014 for ofloxacin, norfloxacin, ciprofloxacin, and enrofloxacin, respectively. Since R_L_ values were lower than unity, the adsorption process can be described as favorable [18].

In real water and wastewater systems, antibiotics are usually present as a mixture instead of individual compounds, and this may affect their adsorption properties. Regarding this concern, isotherm studies were repeated using mixture solutions of fluoroquinolones. The isotherms for fluoroquinolone mixture adsorption by colemanite and Langmuir model fit are given in Figure S4. These results indicate that for the mixture solutions, the adsorption capacities decreased by 18.9%, 10.2%, 16.1%, and 24.3% for ofloxacin, norfloxacin, ciprofloxacin, and enrofloxacin, respectively. These reductions can be attributed to the competition between the target compounds for the adsorption sites.

### 3.3. Effects of pH and Temperature

**Figure 3:**
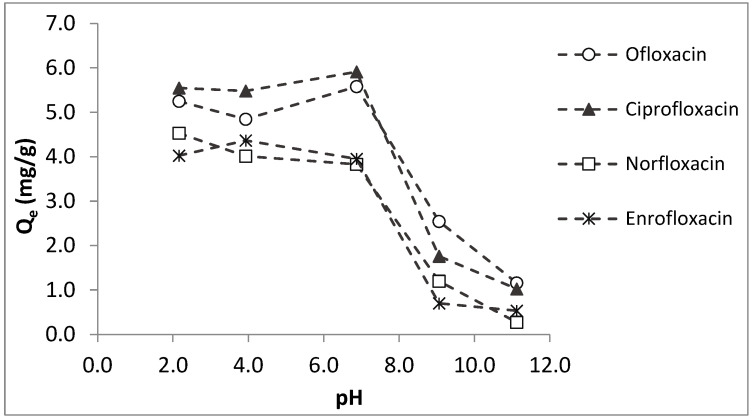
Effects of pH on fluoroquinolone sorption by colemanite (error bars are not shown for clarity).

In order to determine the effects of pH on the adsorption potential of colemanite, equilibrium sorption studies were applied at different solution pH values and the results are given in Figure 3. For the antibiotics studied, the amounts adsorbed per unit weight of colemanite (Qe) are almost constant at acidic and neutral pH values. However, for the pH values higher than 7–7.5, Q_e_ values sharply decrease. These observations can be explained by the interactions of fluoroquinolones with the negatively charged colemanite surface (for the pH range studied). At acidic pH conditions, fluoroquinolones are mainly in cationic forms, in accordance with their pKa_1_ values, i.e., 6.08, 6.34, 6.15, and 6.19 for ofloxacin, norfloxacin, ciprofloxacin, and enrofloxacin, respectively [22,23]. As a result, electrostatic attraction is expected between colemanite and fluoroquinolones at pH values lower than their pKa_1_. For the solution pH between the pKa_1_ and pKa_2_ of fluoroquinolones, the compounds are in their zwitterionic forms. In this case as well, positively charged moieties of fluoroquinolones interact with the negative surface of the colemanite. For the solution pH > pKa_2_, anionic forms of fluoroquinolones dominate, resulting in a repulsion to the negative colemanite surface, which causes lower adsorption in basic pH conditions.

As shown in Figure 3, colemanite adsorption capacity is almost constant for the target fluoroquinolones over a wide range of solution pH. This could be considered an advantage for applicability to real water and wastewaters with different pH values. On the other hand, this is not the case for most adsorbent materials. For example, the norfloxacin adsorption amount using a montmorillonite-biochar composite was low at acidic pH, maximized at neutral conditions, and gradually reduced as the pH increased [13]. In another study, ciprofloxacin adsorption capacity by a montmorillonite-impregnated electrospun cellulose acetate nanofiber was maximized at neutral pH, and the sorption capacity decreased at lower and higher pH values [16].

**Figure 4:**
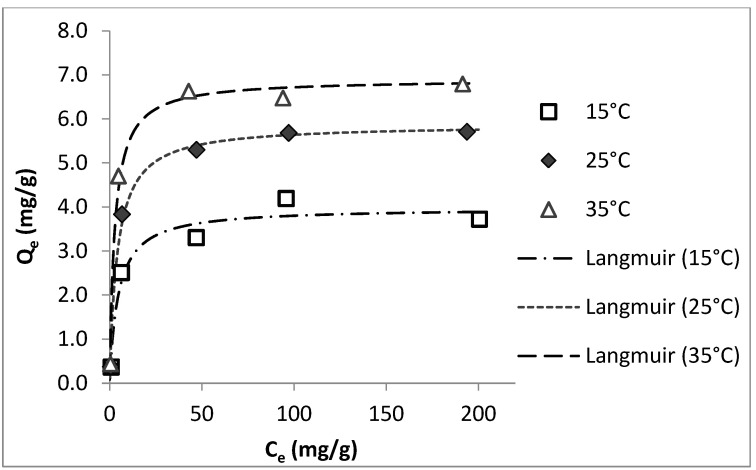
Effects of temperature on ciprofloxacin adsorption by colemanite (error bars are not shown for clarity).

In order to evaluate the temperature effects on the adsorption performance of colemanite, the isotherm experiments were carried out at different temperatures (15, 25, and 35 °C). For these experiments, ciprofloxacin was used as the model compound. The results in Figure 4 show that as the temperature increases, the amount of ciprofloxacin adsorbed by the unit weight of colemanite increases, indicating an endothermic adsorption process. The isotherms at different temperature values fitted well with the Langmuir model. The isotherm model fit results for ciprofloxacin adsorption by colemanite at different temperatures are given in Table S7.

Using the results of equilibrium studies at different temperatures, entropy change (ΔS°), enthalpy change (ΔH°), and Gibbs free energy change (ΔG°) are determined. Equation (3) was used to calculate ΔG°. The dimensionless equilibrium constant (K_c_) was derived from the Langmuir constant (K_L_) using Equation (4), as described in previous studies [18,24]. Then, the van’t Hoff plot (ln K_c_ versus 1/T) was constructed (Figure S5). ΔH° and ΔS° values were determined by the van’t Hoff equation (Equation (5)). The calculated thermodynamic parameters are shown in Table 1.

(3) $$G^{o}=-{\mathrm{RTlnK}}_{C}$$

(4) $$K_{C}=M_{W}\times 55.5\times 1000\times {\;K}_{L}$$

(5) $${\mathrm{lnK}}_{C}=\frac{{\;S}^{ο}}{R}-\frac{{\;H}^{ο}}{\mathrm{RT}}\;$$

where R is the gas constant in J/(mol K), K_c_ is the thermodynamic equilibrium constant (dimensionless), and T is the temperature in K.

The results of thermodynamic studies show that ΔG° is negative for all three temperatures evaluated, suggesting that adsorption is spontaneous. The positive ΔS° value demonstrates increasing randomness at the solid–liquid interface. The positive ΔH° value corresponds to the endothermic process; however, its magnitude is not high (19.81 kJ/mol). Since chemisorption occurs when the adsorption enthalpy change is greater than 200 kJ/mol [25,26], the process in this study can be classified as physical adsorption.

### 3.4. Studies with Real Water and Wastewater

Based on the preliminary studies, the concentrations of the target antibiotics in the water and wastewater samples were below the limits of quantification, i.e., 40–117 ng/L. In order to observe the effects of real water and wastewater matrices, the samples were spiked with a 10 ppm target compound mixture prior to the adsorption experiments. The results for the adsorption in real water and wastewater samples are given in Figure S6. From the obtained results, it can be observed that the amounts adsorbed were slightly lower (up to 8.5%) in tap water compared to those in synthetic water. In surface water, the decrease in the amounts adsorbed was significant (between 38% to 71% reduction). This can be explained by the competing substances in the surface water (e.g., humic acid), resulting in a decrease in the available adsorption sites. When real wastewater was used, the amounts adsorbed were further reduced (59% to 78%) compared to the initial amounts adsorbed. These results indicate that the components in the real wastewater matrix compete for the adsorption sites, leading to reduced adsorption amounts for the target antibiotics.

Various substances in the water and wastewater matrix can compete with fluoroquinolones for adsorption on the colemanite surface. Mainly, compounds having similar functional groups with those of the fluoroquinolones are candidates for such an interaction. Another important factor is the molecular size, where large molecules (such as humic acid) can coat the colemanite surface, causing pore blockage. Moreover, the pK_a_ values of the substances are important in determining their charge states, which are directly related to the adsorption potential of colemanite (this will be further discussed in the Section 3.6).

### 3.5. Desorption and Reuse

**Figure 5:**
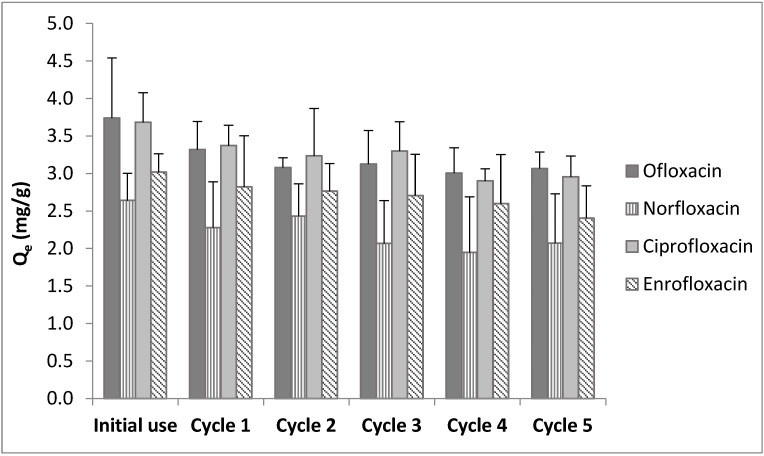
Results of reuse studies.

Desorption and reuse studies were conducted for five cycles of adsorption and desorption by applying 0.1 mol/L NaOH as the eluent solution. Q_e_ values obtained after each cycle are given in Figure 5. The results indicate that the adsorption potential of colemanite was slightly decreased after each reuse experiment.

Based on the results shown in Figure 5, it can be concluded that at the end of five adsorption–desorption cycles, the amounts adsorbed per unit weight of colemanite were mostly retained (i.e., 81.9%, 78.4%, 80.3%, and 79.7% of the initial removal amounts for ofloxacin, norfloxacin, ciprofloxacin, and enrofloxacin, respectively). These results indicate good reuse potential [12,27].

Although adsorption and reuse potentials are important factors, material cost and availability should also be considered when selecting the appropriate treatment alternatives. Colemanite is relatively rare in many areas; however, it is highly available in some regions, such as Turkey, the USA, and Argentina. The unit price of colemanite is 135 EUR/ton. Unfortunately, most of the adsorption studies did not report the material cost (except for a few studies). One of the most commonly used adsorbents is activated carbon (AC), and its unit price ranges from 595 to 4250 EUR/ton, depending on the type, quality, and grade of the material [28]. The alternative adsorbents include montmorillonite (40–120 USD/ton) [28] and biochar (350–1200 USD/ton) [29]. From these data, it can be inferred that the colemanite price is much lower than AC, and it is in a comparable range with other alternative adsorbents.

### 3.6. Adsorption Mechanism

**Figure 6:**
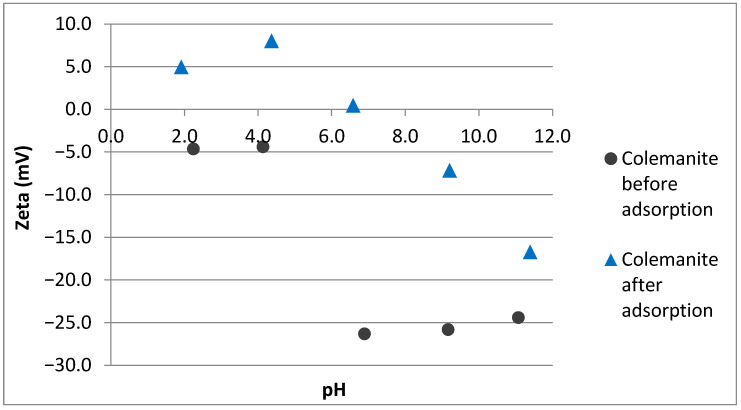
pH versus zeta potential of colemanite before and after adsorption.

The adsorption mechanism for fluoroquinolone antibiotics by colemanite was elucidated by material characterization before and after adsorption. pH versus zeta potential plots of colemanite (before and after adsorption) are given in Figure 6. Based on this figure, it can be observed that colemanite has a negative surface charge for the pH range used in this study. After the adsorption process, the surface is positively charged at low pH values, with an isoelectric point (IEP) at around 6.6. This result indicates that the surface becomes more positively charged after the adsorption process, which can be explained by the positively charged amine groups of the adsorbate molecules attracted by the negative colemanite surface.

The SEM image and EDS analysis of colemanite after adsorption are given in Figure S7a and b, respectively. The SEM image indicates that the surface becomes less porous after adsorption, which can be explained by the pore-filling mechanism during the adsorption process. According to the EDS analysis results, the percentage of boron content on the colemanite surface was slightly reduced (about 2.5%), while carbon percent was increased (1.6%), and trace amounts of nitrogen and fluorine were present. These results indicate that fluoroquinolones were successfully adsorbed on the colemanite surface.

**Figure 7:**
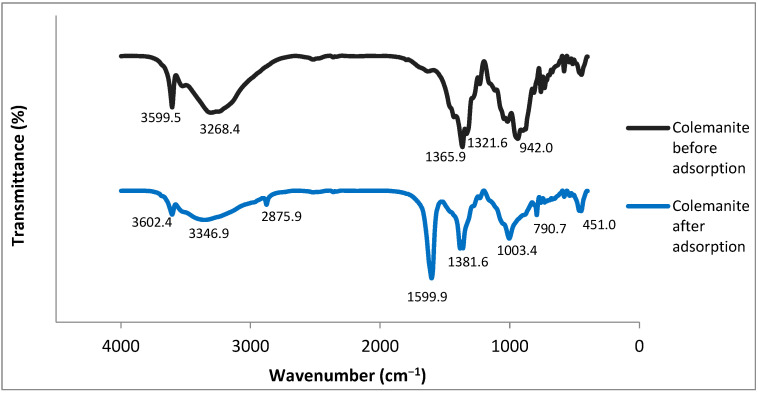
FTIR spectra of colemanite before and after adsorption of fluoroquinolones.

FTIR spectra for colemanite before and after adsorption (Figure 7) have been interpreted according to previous studies [30,31]. The bands at 3326.91 and 3609.50 cm^−1^ correspond to the O–H stretching vibrational peaks present in the colemanite structure. The peak at 1684.14 cm^−1^ represents the bending vibration of H–O–H, indicating a crystalline water presence. The bands at 1373.01 and 1318.77 cm^−1^ can be explained by the asymmetric stretching of three-coordinate boron. The peak at 1224.57 cm^−1^ depicts the bending vibrations of B–OH. Symmetric and asymmetric stretching bands of tetra-coordinated boron are observed at 762.14 and 963.39 cm^−1^. The band at 916.29 cm^−1^ is due to three-coordinate boron symmetric stretching. The final peaks (<725 cm^−1^) represent the bending vibrations of the borate units.

Significant differences can be observed for the FTIR spectra of colemanite before and after adsorption. For the FTIR spectra of colemanite after adsorption, new bands at 2871.62 cm^−1^ and 1611.36 cm^−1^ appeared. The band at 2871.62 cm^−1^ can be attributed to the C–H stretching vibrations, and the band at 1611.36 cm^−1^ is assigned to N–H bending vibrations, indicating the presence of quinolone antibiotics [32]. It is known that in the 3700–2500 cm^−1^ region, O–H and N–H frequencies are present, and hydrogen bonding influences the peak shapes [33]. For colemanite before adsorption, the bands at 3609.50 and 3326.91 cm^−1^ were moved to 3615.21 and 3386.85 cm^−1^, respectively, and broadened after adsorption. These changes indicate the presence of intermolecular hydrogen bonding, which can be explained by the interactions between the O–H groups of colemanite and the O–H and N–H groups of the fluoroquinolones. Moreover, after the adsorption process, the band corresponding to the bending of B–OH (1224.57 cm^−1^) was moved to 1218.86 cm^−1^, and it became narrower. The symmetric and asymmetric stretching vibrations of tetra-coordinated boron were shifted to 793.54 and 1017.62 cm^−1^, respectively. The asymmetric stretching bands of three-coordinate boron were shifted from 1373.01 and 1318.77 cm^−1^ to 1390.13 and 1381.57 cm^−1^, respectively. These wavenumbers are close to the range of vibrations of the protonated amine group in the piperazine moiety of fluoroquinolones (1400 cm^−1^) [34,35]. As a result, the changes after the adsorption process can be explained by the interactions of negatively charged borate units with the protonated amine groups of fluoroquinolone antibiotics.

Based on the adsorption mechanism discussed above, it can be deduced that pollutants with similar physicochemical properties to those of fluoroquinolones can be removed by colemanite from aqueous streams. As indicated in the zeta potential analysis results, colemanite has a negative surface charge over a wide pH range, and it can attract compounds with positively charged moieties. For example, compounds with amino functional groups (i.e., several dyes and pharmaceutically active compounds) are suitable for this adsorption mechanism. However, for a more precise estimation, pK_a1_ and pK_a2_ values of the target compounds should be evaluated together with their available surface functional groups that can interact with colemanite, which is considered a future research perspective.

## 4. Conclusions

In this study, colemanite was used as an alternative adsorbent for the removal of fluoroquinolone antibiotics from water and wastewater. Colemanite was characterized by SEM/EDS, specific surface area, zeta potential, XRD, and FTIR analyses. Systematic batch adsorption experiments were conducted, and the PSO model described well the kinetic results. The maximum adsorption capacities obtained by the Langmuir model were 5.19, 3.43, 5.88, and 3.69 mg/g for ofloxacin, norfloxacin, ciprofloxacin, and enrofloxacin, respectively. Adsorption studies at different pH values demonstrated that the amount of fluoroquinolones adsorbed by colemanite was stable for acidic and neutral environments, but it started to decrease at basic pH values. Thermodynamic studies suggested that the adsorption was spontaneous and endothermic, with increased randomness at the solid–liquid interface during the adsorption process. Studies with real water and wastewater samples indicated reductions in the fluoroquinolone adsorption amounts (up to 78%). Five adsorption–desorption cycles were applied to test the reusability of colemanite, and the sorption capacity was highly maintained (78.4–81.9%). Based on the material characterization before and after adsorption experiments, the adsorption mechanism is explained by the electrostatic attraction between protonated amine groups of fluoroquinolones and the negatively charged borate units of colemanite.

## Figures and Tables

**Table 1:** Thermodynamic parameters derived for the adsorption of ciprofloxacin by colemanite.

Temperature (°C)	ΔG° (kJ/mol)	ΔH°(kJ/mol)	ΔS° (kJ/mol)
15	−36.36	19.81	0.19
25	−37.90		
35	−40.27		

## Data Availability

The data presented in this study are available upon request from the corresponding author.

## Supplementary Materials

The following supporting information can be downloaded at: https://www.mdpi.com/article/10.3390/ijerph20032646/s1, Table S1: Experimental conditions used in adsorption and desorption studies, Table S2: LC-MS/MS parameters for the target antibiotics; Figure S1: Nitrogen adsorption–desorption isotherm of colemanite; Figure S2: SEM image (a) and EDS analysis (b) of colemanite; Figure S3: XRD analysis of colemanite; Table S3: Kinetic and isotherm models applied to the experimental data; Table S4: Parameters used for model evaluation; Table S5: Kinetic model fit results; Table S6: Isotherm model fit results; Table S7: Isotherm model fit results for ciprofloxacin adsorption by colemanite at different temperatures; Figure S4: Isotherms for fluoroquinolone mixture adsorption by colemanite and Langmuir model fit; Figure S5: The van’t Hoff plot; Figure S6: Adsorption in real water and wastewater samples; Figure S7: SEM image (a) and EDS analysis (b) of colemanite after adsorption.
