# Supplementary material for: Adsorption of Fluoroquinolone Antibiotics from Water and Wastewater by Colemanite
Source: Int J Environ Res Public Health. 2023 Feb 1;20(3):2646. doi: 10.3390/ijerph20032646 (PMC9915184; doi:10.3390/ijerph20032646)
Supplement: Supplementary file 1 [file ijerph-20-02646-s001.zip › ijerph-2139758-supplementary.docx]

Supplementary material

Adsorption of fluoroquinolone antibiotics from water and wastewater by colemanite

Gül Gülenay Hacıosmanoğlu^a^ ^*^, Marina Arenas^b^, Carmen Mejías^b^, Julia Martín^b^, Juan Luis Santos^b^, Irene Aparicio^b^, Esteban Alonso^b^

^a^ Environmental Engineering Department, Faculty of Engineering, Marmara University,

Uyanık Cd. No:6, 34840 Istanbul, Turkey

^b^ Departamento de Química Analítica, Escuela Politécnica Superior, Universidad de Sevilla, C/Virgen de África, 7, E-41011 Seville, Spain

^*^ Corresponding Author

Address:

Marmara University

Environmental Engineering Department

Uyanık Cd. No:6, 34840

Istanbul, Turkey

Tel.: +90 216 777 36 13 ext 3613; Fax: +90 216 348 0293.

E-mail: g.haciosmanoglu@gmail.com (Gül Gülenay Hacıosmanoğlu)

Table S1. Experimental conditions used in adsorption and desorption studies ^*^

| **Experiment type** | **Contact time (min)** | **Initial adsorbate concentration (mg/L)** | **Temperature (°C)** | **Adjusted**  **pH** ^**^ |
| --- | --- | --- | --- | --- |
| Adsorption kinetics | 5-10,080 | 10 | 25 | no pH adjustment |
| Adsorption isotherms | 1440 | 1-200 | 25 | no pH adjustment |
| Effects of temperature | 1440 | 10 | 15, 25, 35 | no pH adjustment |
| Effects of pH | 1440 | 10 | 25 | 2, 4, 6, 9, 11 |
| Experiments with water and wastewater samples | 1440 | 10 | 25 | no pH adjustment |
| Desorption and reuse | 1440 | 10 | 25 | no pH adjustment |

^*^ The constant parameters were mixing speed (350 rpm), solution volume (10 ml) and adsorbent dose (1 g/L).

^**^ In the experiments with no pH adjustment, the solution pH was about 6.8.

**Table S2.** LC–MS/MS parameters for the target antibiotics.

| **Compound** | **Ionization mode** | **Precursor ion (*m/z*)** | **Product ions (MRM1/MRM2 *(m/z)*** | **Fragmentor (V)** | **Collision energy (V)** | **Retention time (min)** |
| --- | --- | --- | --- | --- | --- | --- |
| Ciprofloxacin | Positive | 332.1 | 314.1/231.0 | 166 | 16/40 | 4.2 |
| Norfloxacin | Positive | 320.1 | 302.1/276.2 | 166 | 24/16 | 3.8 |
| Ofloxacin | Positive | 362.4 | 318.2/261.2 | 166 | 20/32 | 3.5 |
| Enrofloxacin | Positive | 360.4 | 286.1/342.1 | 166 | 40/40 | 4.6 |


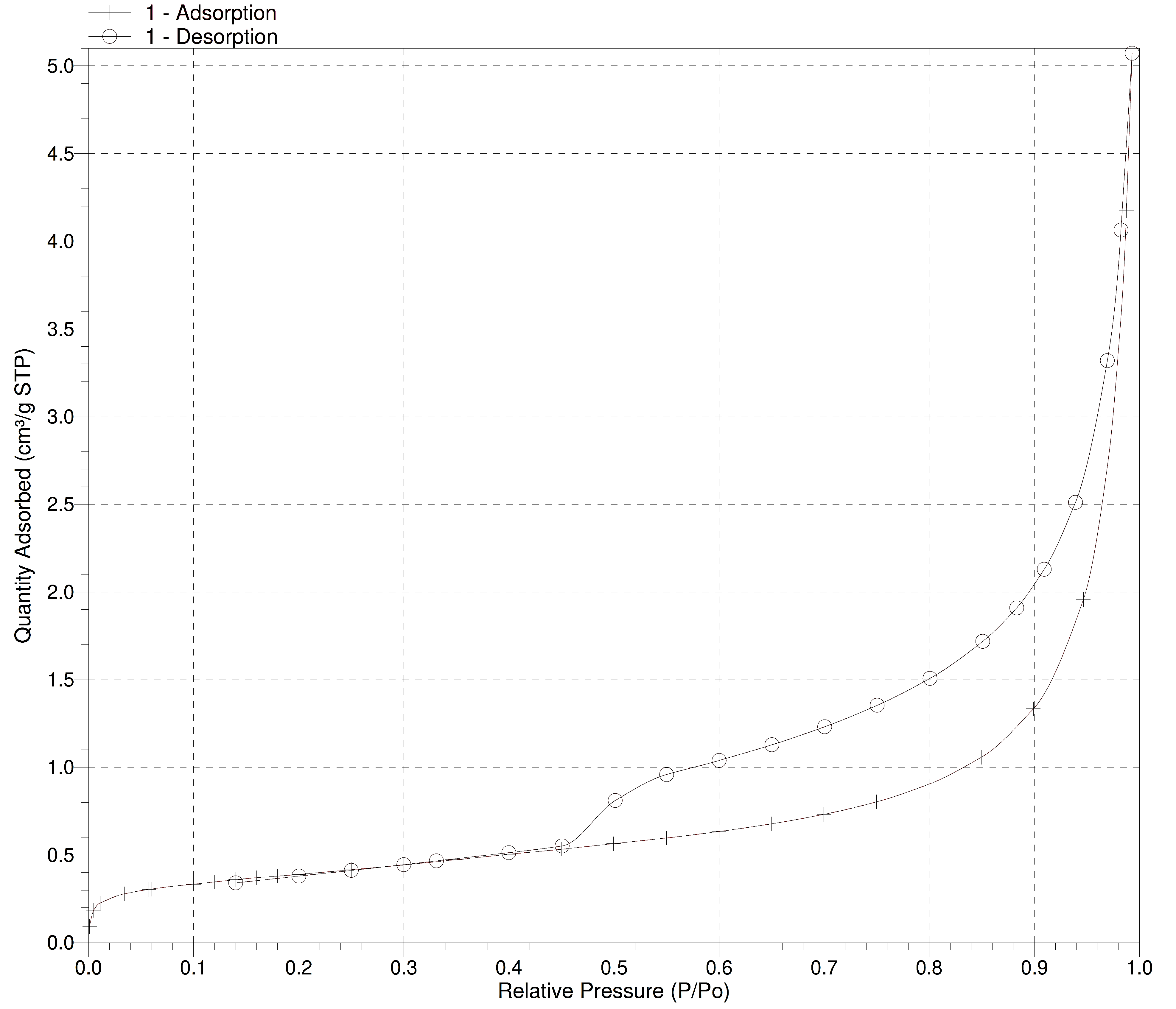


**Figure S1.** Nitrogen adsorption-desorption isotherm of colemanite.


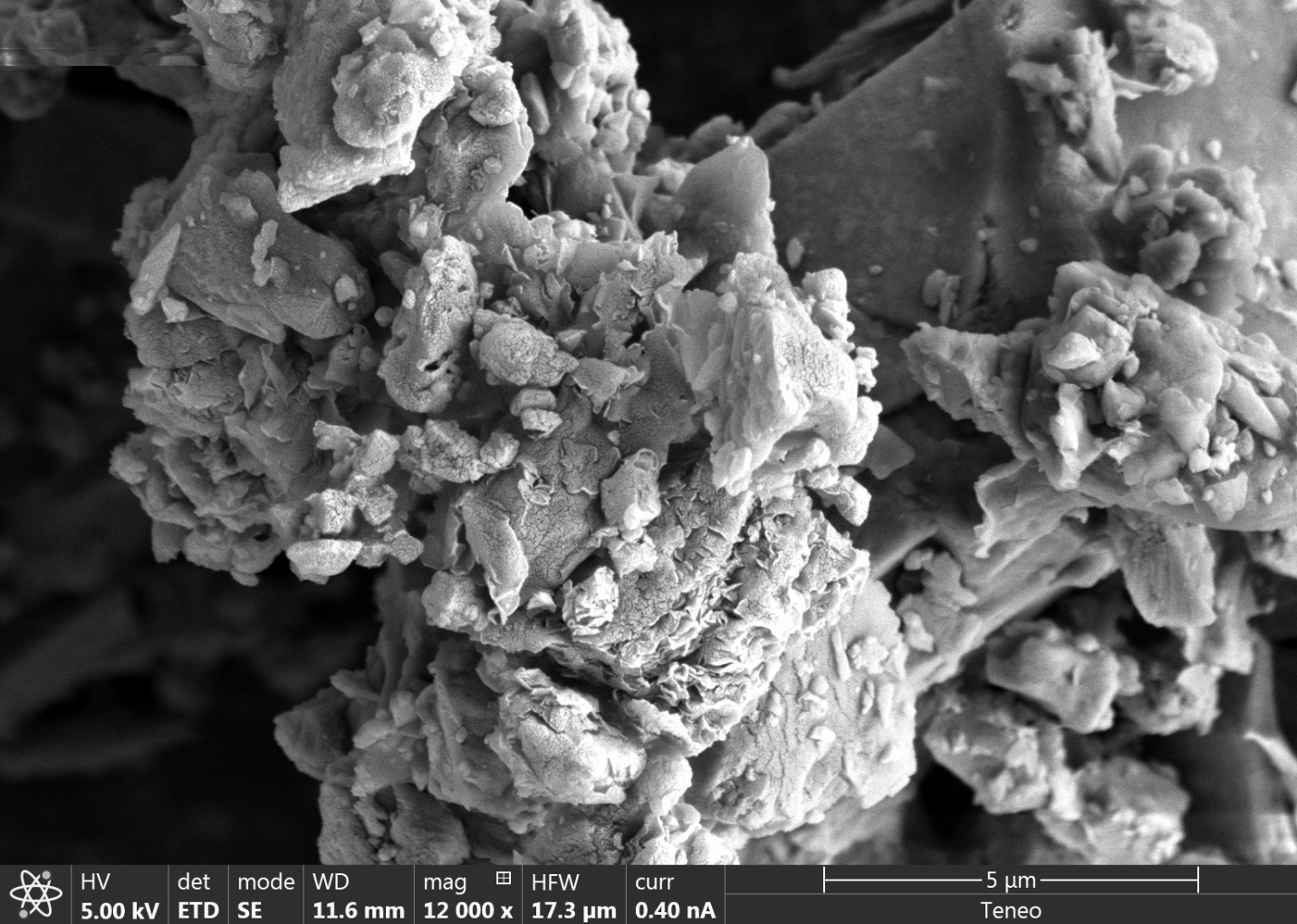


(a)

(b)

**Figure S2.** SEM image (a) and EDS analysis (b) of colemanite.


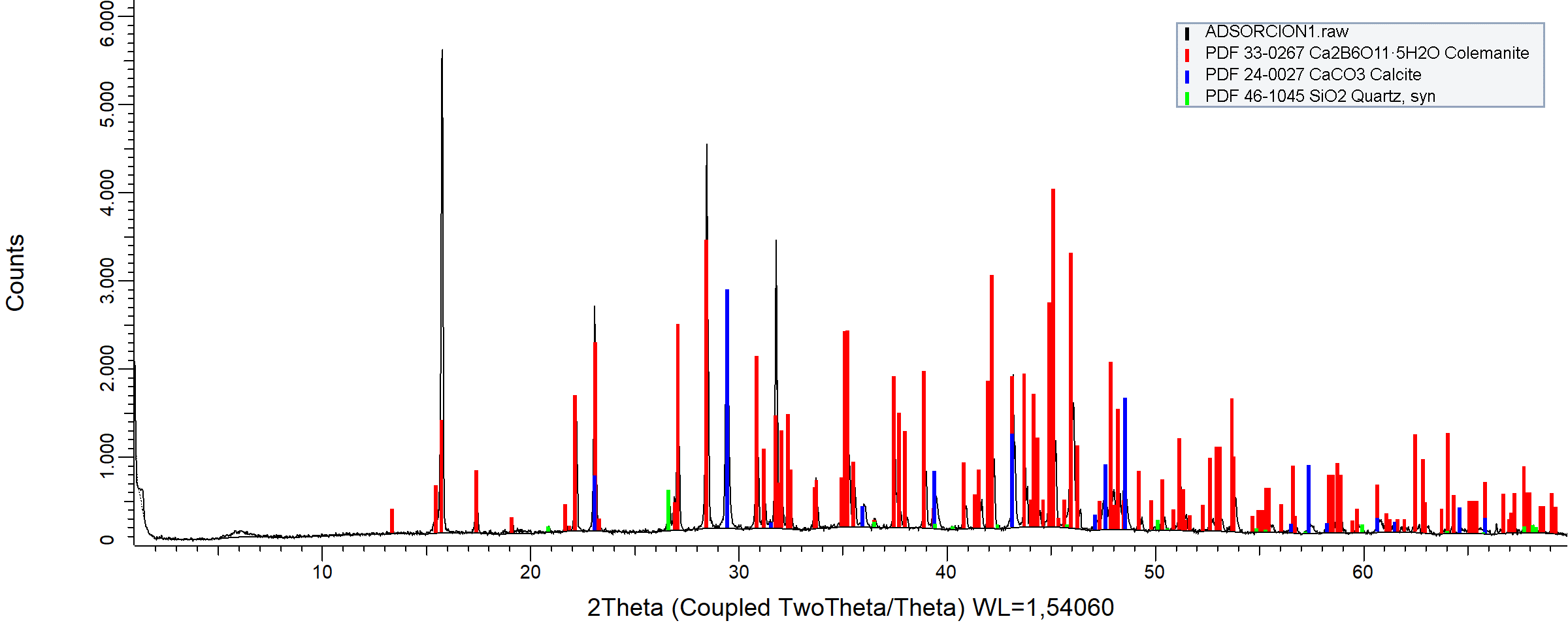


**Figure S3.** XRD analysis of colemanite.

**Table S3.** Kinetic and isotherm models applied to the experimental data.

| **Model Type** | **Model Name** | **Equation** |
| --- | --- | --- |
| **Kinetic** | **Pseudo-first order (PFO)** | $q_{t}= q_{e}\left( 1- e^{-k_{1}t} \right)$ |
|  | **Pseudo-second order (PSO)** | $q_{t}=\frac{q_{e}^{2}k_{2}t}{1+q_{e}k_{2}t}$ |
|  | **Elovich** | $q_{t}=\frac{1}{\beta}\ln(1+\alpha\beta t)$ |
| **Isotherm** | **Langmuir** | $q_{e}=\frac{{Q_{max}K_{L}C}_{e}}{{1+ K_{L}C}_{e}}$ |
|  | **Freundlich** | $q_{e}=K_{F}C_{e}^{n}$ |

where, q_t_ and q_e_ are the amounts of pollutant adsorbed (mg/g) at time, t (min) and at equilibrium; k_1_ is the rate constant of pseudo-first order equation (1/min); k_2_ is the rate constant of pseudo-second order equation (g/(mg⋅min)); α and β are Elovich constants; C_e_ (mg/L) is the equilibrium concentration of pollutant; Q_max_ (mg/g) is the maximum adsorption capacity calculated from Langmuir model; K_L_ (L/mg) is the Langmuir constant; K_F_ ((mg/g)/(mg/L)^n^) is the Freundlich constant; n (dimensionless) is the Freundlich intensity parameter.

**Table S4.** Parameters used for model evaluation.

| **Parameter** | **Equation** |
| --- | --- |
| Chi square | $\chi^{2}=\sum_{i=1}^{n} \frac{{(q_{\exp}-q_{\mathrm{calc}})}^{2}}{q_{\mathrm{calc}}}$ |
| Correlation coefficient | $R^{2}=1- \frac{\sum_{i=1}^{n} {(q_{\exp}-q_{\mathrm{calc}})}^{2}}{\sum_{i=1}^{n} {(q_{\exp}-q_{exp, mean})}^{2}}$ |
| Normalized root mean square error | $NRMSE= \frac{\sqrt{\frac{\sum_{i=1}^{n} {(q_{\exp}-q_{\mathrm{calc}})}^{2}}{n}}}{q_{exp,max}-q_{exp, min}}$ |

Where n is the number of data points; q_exp_ and q_calc_ are the quantities adsorbed determined experimentally and the quantities adsorbed calculated by the models, respectively; q_exp,max_ and q_exp,min_, q_exp,mean_ are the maximum, minimum and mean values of the quantities adsorbed determined experimentally, respectively.

**Table S5.** Kinetic model fit results.

| **Antibiotic** | **Model** | **Model Parameters** | **R^2^** | **χ^2^** | **NRMSE** |
| --- | --- | --- | --- | --- | --- |
| **Ofloxacin** | **PFO** | k_1_ = 0.082 min^-1^ q_e_ = 3.2 mg/g | 0.814 | 0.3326 | 0.1311 |
|  | **PSO** | k_2_ = 0.035 g/(mg×min) q_e_ = 3.352 mg/g | 0.901 | 0.1948 | 0.0955 |
|  | **Elovich** | α = 45.7 mg/(g×min) | 0.801 | 0.4951 | 0.1358 |
|  |  | β = 3.693 g/mg |  |  |  |
| **Norfloxacin** | **PFO** | k_1_ =0.084 min^-1^ q_e_ = 2.584 mg/g | 0.777 | 0.3298 | 0.1391 |
|  | **PSO** | k_2_ = 0.049 g/(mg×min) q_e_ = 2.698 mg/g | 0.846 | 0.2033 | 0.1157 |
|  | **Elovich** | α = 59.7 mg/(g×min) | 0.781 | 0.3747 | 0.138 |
|  |  | β = 4.757 g/mg |  |  |  |
| **Ciprofloxacin** | **PFO** | k_1_ = 0.050 min^-1^ q_e_ = 3.310 mg/g | 0.829 | 0.857 | 0.149 |
|  | **PSO** | k_2_ = 0.026 g/(mg×min) q_e_ = 3.409 mg/g | 0.920 | 0.2529 | 0.1017 |
|  | **Elovich** | α = 26.8 mg/(g×min) | 0.763 | 0.549 | 0.1753 |
|  |  | β = 3.551 g/mg |  |  |  |
| **Enrofloxacin** | **PFO** | k_1_ = 0.073 min^-1^ q_e_ = 3.086 mg/g | 0.941 | 0.1557 | 0.0828 |
|  | **PSO** | k_2_ = 0.036 g/(mg×min) q_e_ = 3.207  mg/g | 0.936 | 0.1550 | 0.0862 |
|  | **Elovich** | α = 79.1 mg/(g×min) | 0.683 | 0.7529 | 0.1915 |
|  |  | β = 4.126 g/mg |  |  |  |

**Table S6.** Isotherm model fit results.

| **Adsorbate** | **Model** | **Model Parameters** | **R^2^** | **χ^2^** | **NRMSE** |
| --- | --- | --- | --- | --- | --- |
| **Ofloxacin** | **Langmuir** | Q_max_ = 5.19 mg/g K_L_ = 0.369 L/mg | 0.968 | 0.336 | 0.067 |
|  | **Freundlich** | K_F_ = 2.080 (mg/g)/(mg/L)^n^  n = 1.883 | 0.711 | 1.786 | 0.200 |
| **Norfloxacin** | **Langmuir** | Q_max_ = 3.43 mg/g K_L_ = 0.407 L/mg | 0.923 | 0.357 | 0.099 |
|  | **Freundlich** | K_F_ = 1.385 (mg/g)/(mg/L)^n^  n = 0.188 | 0.699 | 1.228 | 0.194 |
| **Ciprofloxacin** | **Langmuir** | Q_max_ = 5.88 mg/g K_L_ = 0.240 L/mg | 0.989 | 0.218 | 0.039 |
|  | **Freundlich** | K_F_ = 1.940 (mg/g)/(mg/L)^n^  n = 0.227 | 0.826 | 1.494 | 0.158 |
| **Enrofloxacin** | **Langmuir** | Q_max_ = 3.69 mg/g K_L_ = 0.361 L/mg | 0.920 | 0.392 | 0.105 |
|  | **Freundlich** | K_F_ = 1.460 (mg/g)/(mg/L)^n^  n = 0.193 | 0.697 | 1.324 | 0.204 |

**Table S7.** Isotherm model fit results for ciprofloxacin adsorption by colemanite at different temperatures.

| **Temperature** | **Model** | **Model Parameters** | **R^2^** | **χ^2^** | **NRMSE** |
| --- | --- | --- | --- | --- | --- |
| 15 °C | Langmuir | Q_max_ = 3.982 mg/g K_L_ = 0.214 L/mg | 0.954 | 0.222 | 0.075 |
|  | Freundlich | K_F_ = 1.287 (mg/g)/(mg/L)^n^  n = 0.228 | 0.802 | 0.964 | 0.156 |
| 25 °C | Langmuir | Q_max_ = 5.88 mg/g K_L_ = 0.240 L/mg | 0.989 | 0.218 | 0.039 |
|  | Freundlich | K_F_ = 1.940 (mg/g)/(mg/L)^n^  n = 0.227 | 0.826 | 1.494 | 0.158 |
| 35 °C | Langmuir | Q_max_ = 6.903 mg/g K_L_ = 0.368 L/mg | 0.984 | 0.343 | 0.048 |
|  | Freundlich | K_F_ = 2.587 (mg/g)/(mg/L)^n^  n = 0.2039 | 0.788 | 2.063 | 0.174 |

**Figure S4.** Isotherms for fluoroquinolone mixture adsorption by colemanite and Langmuir model fit.

**Figure S5.** van't Hoff plot.

**Figure S6.** Adsorption in real water and wastewater samples.


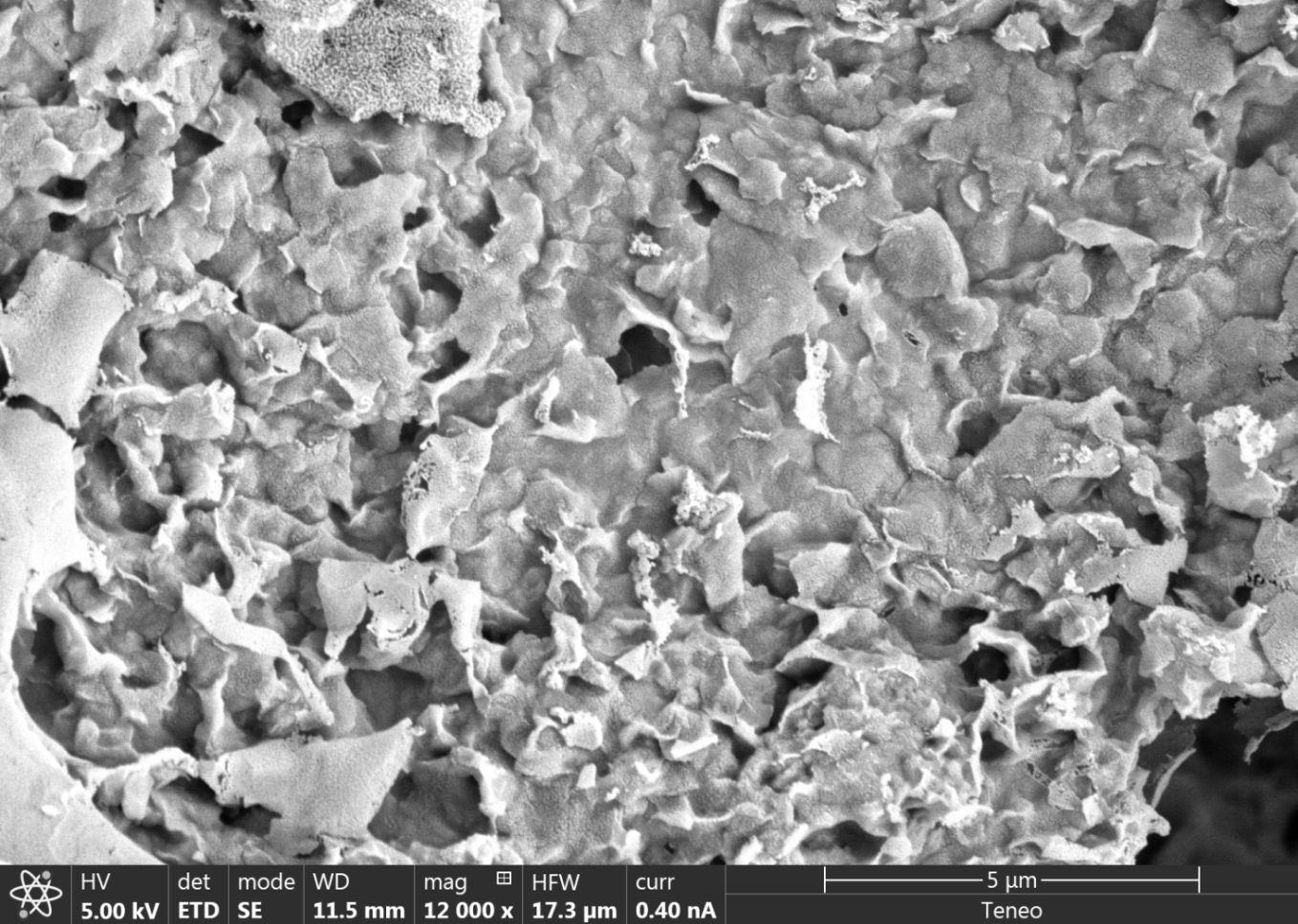


(a)

(b)

**Figure S7.** SEM image (a) and EDS analysis (b) of colemanite after adsorption.
